# BSA modification of bacterial surface: a promising anti-cancer therapeutic strategy

**DOI:** 10.1186/s12866-023-02830-z

**Published:** 2023-04-17

**Authors:** Zhongming He, Kun Chen, Yu An, Jie He, Xiaoli Zhang, Lannan Tang, Fa Sun, Kehua Jiang

**Affiliations:** 1 Guizhou Medical University, Guiyang, China; 2 Department of Urology, Guizhou Provincial People’s Hospital, Guiyang, China; 3 Department of Medical Genetics, Guizhou Provincial People’s Hospital, Guiyang, China; 4 NHC Key Laboratory of Pulmonary Immune-Related Diseases, Guizhou Provincial People’s Hospital, Guiyang, China; 5 College of Medical, Guizhou University, Guizhou, 550000 China

**Keywords:** *Escherichia coli* (*E. coli*), Bovine serum albumin (BSA), Surface modification, Anti-cancer

## Abstract

**Background:**

Attenuated live bacterial therapy and medical BSA materials have their own advantages in anti-cancer research, and their combination is expected to overcome some of the disadvantages of conventional anti-cancer therapeutics.

**Methods and objective:**

Utilizing the high affinity between biotin and streptavidin, BSA modification on the surface of *Escherichia coli* (*E. coli*) was achieved. Then, the adhesion and targeting abilities of BSA modified *E. coli* was explored on different bladder cancer cells, and the underlying mechanism was also investigated.

**Results:**

BSA modification on the surface of *E. coli* enhances its ability to adhere and target cancer cells, and we speculate that these characteristics are related to the expression of SPARC in different bladder cancer cell lines.

**Conclusion:**

BSA and live bacteria have their own advantages in anti-cancer research. In this study, we found that *E. coli* surface-modified by BSA had stronger adhesion and targeting effects on bladder cancer cells with high expression of SPARC. These findings pave the way for the future studies exploring the combination of BSA combined with live bacteria for cancer therapy.

**Supplementary Information:**

The online version contains supplementary material available at 10.1186/s12866-023-02830-z.

## Introduction

Cancer is a major global public health problem. Conventional anti-cancer methods have failed to reduce the increasing mortality rate of cancer every year. It has also revealed many disadvantages such as non-specific effects on normal cells, low bioavailability, cytotoxicity and lack of effect on metastasis [1–3]. In recent years, in order to treat cancer more effectively, many new anti-cancer therapies and drug delivery strategies, including micro/nanoparticles [4], liposome [5], solid lipid nanoparticles (SLNS) [5, 6], polymer nanoparticles [7], dendritic macromolecules [8]^.^and micelles [9], have been developed and shown good efficacy. However, all the above strategies have some limitations, such as low stability in vivo, leakage during transport, incomplete local targeting, and inability to target deeper parts of the tumor [10].

With the advancement of medical materials, BSA has rapidly garnered much research attention. As a biocompatible, degradable, non-toxic and sustainable material, BSA can form complexes with poorly soluble or exogenous substances and assist in substance transport [11–13]. As many tumors express high levels of BSA-binding proteins (e.g., SPARC), it is often loaded with Polymer Materials as an anti-cancer targeted drug delivery system [14–16]. These characteristics of BSA largely overcome the shortcomings of previous studies. Owing to its high aqueous solubility, simple preparation and rich functional groups, BSA has emerged as one of the most promising medical polymer materials in the fields of drug carriers, surface modifiers and biomimetic templates [14, 17]. However, free BSA is often complicated by its instability in aqueous media, which may lead to its degradation and partial loss of physicochemical properties [18, 19]. In order to solve this problem, previous studies combined BSA with inorganic molecular materials [12, 20]. Although this solved the issue of stability of the aqueous solution of BSA materials, it also reduced its biocompatibility and biodegradability to varying degrees [21–23]. In addition, the complex human immune system, vasculature, low pH, high interstitial fluid pressure in the tumor immune microenvironment (TME), and the cytoplasmic matrix released from the microenvironment have all become protection and escape barriers for tumor cells. These barriers make it difficult for drugs to reach the hypoxic and necrotic core area of tumors, thereby reducing drug utilization and causing more serious side effects [24, 25].

Regarding the above issue, we focused on live attenuated bacteria with good biocompatibility, immunogenicity, ideal adjuvant and autotoxicity characteristics and great anti-cancer potential [26, 27]. Compared with non-biological materials, live attenuated bacteria not only serve as carriers for delivering therapeutic agents to tumor sites, but also interact with the immune system and stimulate it to recognize and destroy tumor cells [28]. Theoretically, bacteria can enter the host through various routes such as intravenous, subcutaneous and intratumoral injection, and disperse in various parts of the body, including normal organs and solid tumors [29]. Normally, the number of bacteria distributed in normal tissues, organs and vasculature sharply drops within a short period due to the oxygen-enriched physiological environment of the body and the immune system, and is eventually permanently eliminated to avoid potential toxicity to the host [30, 31]. Bacteria that initially reach solid tumors migrate to the hypoxic/necrotic core area within the tumor. The nutrients and hypoxic environment in the hypoxic/necrotic core area of the tumor further promotes the proliferation of anaerobic bacteria [32, 33]. At the same time, the immunosuppressive tumor microenvironment also prevents early recognition and clearance of therapeutic bacteria by the immune system. Eventually, the constantly multiplying bacteria continues to activate the host's immune system, causing a large number of immune cells to infiltrate into the tumor and kill tumor cells [34]. Dead tumor cells continue to release nutrients for the sustained growth and reproduction of the bacteria. In addition, the characteristics of easy genetic manipulation also allow their programming to synthesize and secrete anti-cancer drugs, and changing their metabolic pathways to improve the secretion of therapeutic toxin proteins and bacterial derivatives related to tumor treatment [35–37]. Abundant levels of pathogen-associated molecular patterns (PAMPs) enable the bacteria to effectively activate immune cells, enabling the recognition of tumor cells by specific immune cells and elimination of tumor cells even in the tumor immunosuppressive microenvironment, triggering the tumor specific immune response and minimal side effects [38, 39].

In this study, we exploited the interaction between biotin and streptavidin to couple streptavidin-labeled BSA to the surface of biotinylated *E. coli*, resulting in BSA modification on the surface of *E. coli*. While addressing the poor aqueous solubility of free BSA and maintaining the desired biocompatibility, biodegradability and other physical and chemical properties of the composite material, the respective targeting capabilities of bacteria and BSA were also jointly enhanced. Based on these results, we further evaluated the adhesion and tumor targeting ability of *E. coli*-BSA on bladder cancer cells and the associated mechanism, laying the foundation for future studies exploring the combination of BSA with live bacteria therapy for treating cancer.

## Materials and methods

### Materials

*E. coli* (BL21) was a generous gift from Professor Yu Fuxun, Central Laboratory of Guizhou people's Hospital; Streptavidin Coupling Kit-Lightning-Link® was purchased from Abcam, ab102921; Streptavidin Alexa Fluor ®488Monovalent antibody Labeling Kit was obtained from Abcam, ab272187; EZ-Link 'Sulfo-NHS- Biotin was procured from Thermo Fisher, 2721; LIVE/DEAD' BacLight 'Bacterial Viability Kit was purchased from Thermo Fisher Scientific; Propidium iodide was obtained from GLPBIO; Human SPARC ELISA Kit was purchased from Boster, Wuhan; Glutaraldehyde, 2.5% (EM Grade); Bovine Serum Albumin (BSA); pET-28a-EGFP-C was purchased from YouBio, VT8025; Cell Plasma Membrane Staining Kit with DiI, Actin-Tracker Red-555 was purchased from Beyotime. Streptavidin; and mounting medium, antifading (with DAPI), DAPI solution (10ug/ml), *E. coli* BL21(DE3) Competent Cells and Coomassie brilliant Blue were purchased from Beijing Solaibao Technology (Beijing, China).

### Research methods

**Fig. 1:**
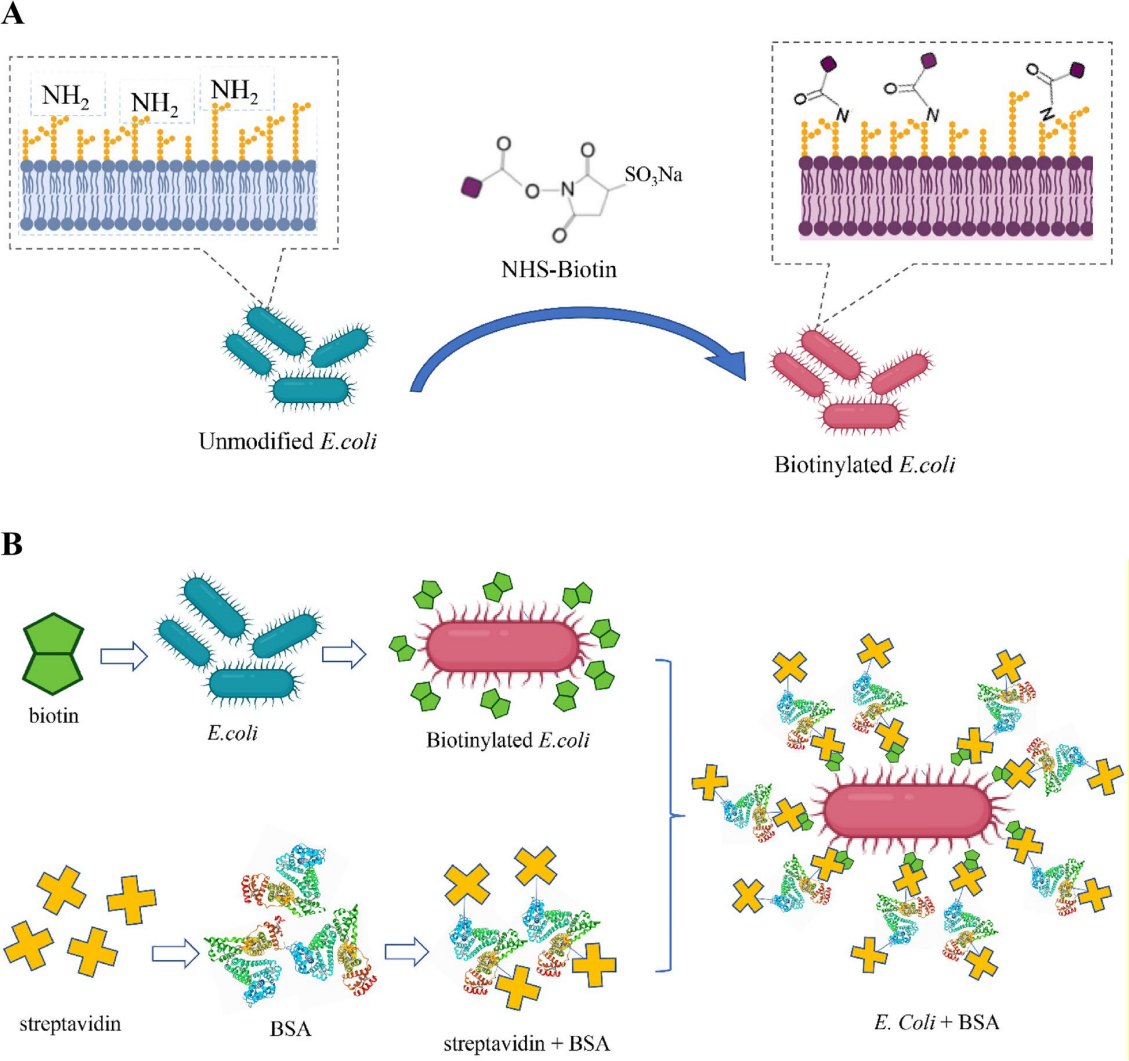
**A** Schematic diagram showing the biotinylation of *E. coli* surface, where primary amines on the membrane reacted with N-hydroxysulfosuccinimide, forming amide bonds between the *E. coli* surface and biotin. **B** The Process of BSA targeting *E. coli.* Figures were generated with the help of BioRender (biorender.com)

BSA modified *E. coli* synthesis was divided into three steps. Firstly, biotin was conjugated to the surface of the bacteria using N-hydroxysuccinimide ester (NHS), which reacted with ubiquitous primary amines on the bacterial surfaces (Fig. 1A). Secondly, streptavidin was coupled to the surface of BSA using Streptavidin coupling kit (Abcam, ab102921). Finally, through the specific reaction between biotin and streptavidin, biotinylated *E. coli* was combined with the BSA-streptavidin to complete the surface BSA modification of *E. coli* (Fig. 1B).

#### Biotinylation of *E. coli* surface

*E. coli* cultures were inoculated from a glycerol stock. Before use, a single colony from LB solid medium was cultured in a conical flask containing 100 ml LB liquid medium, and placed on a shaker (37℃, 190 rpm) until the bacterial growth reached the logarithmic phase. The bacterial liquid was taken at the logarithmic phase, centrifuged for 5 min at 3600 rpm and washed thrice with ice-cold phosphate buffered saline (PBS). *E. coli* was then biotinylated with sulfo-NHS-functionalized biotin (EZ-Link Sulfo-NHS-Biotin, ThermoFisher) with 1 mg sulfo-NHS biotin per mL of *E. coli* culture. All biotinylation reactions were carried out with *E. coli* when OD600 was 1.0. The reaction lasted for 30 min on ice. After biotinylation, the bacterial suspension of *E. coli* was centrifuged and washed thrice with sterile PBS.

##### Detection of biotinylated *E. coli*

All the biotinylated species were incubated with a 1:100 dilution of a fluorescent streptavidin conjugate (Streptavidin Alexa Fluor® 488 (Monovalent); ab272187). After incubating at room temperature for 30 min, Triton x-100 (0.02% solarbio, Beijing) and Glutaraldehyde (2.5% EM Grade) were added to *E. coli* for 20 min, respectively. The red-fluorescent DNA stain, propidium iodide (PI), was added to co-localize with the green fluorescent streptavidin. Then the solution was washed thrice with PBS for 5 min each time. *E. coli* was examined and imaged by fluorescence microscope (Olympus) after standing at room temperature for 1 h.

#### Preparation of streptavidin-conjugated BSA

Streptavidin-conjugated BSA was prepared according to the manufacturer’s instructions (Abcam, ab102921). 100 µL of BSA(1 mg/mL) was taken to be labeled, 10 µL modifier reagent, was added and the above mixture was added to streptavidin mix and gently mixed and incubated for overnight. Then 10µL Quencher reagent was added for every 100µL of BSA in the reaction and incubated at 20 °C for 30 min before use. Then the formation of Streptavidin-conjugated BSA was confirmed by denaturing non-reducing protein gel electrophoresis and Coomassie brilliant blue staining.

#### Preparation of biotinylated *E. coli* linked to BSA

50µL biotinylated E. coli was mixed with 50µL streptavidin-labeled BSA, incubated on ice with constant stirring for 30 min and washed twice with sterile PBS to obtained biotinylated *E. coli* modified by BSA.

##### Detection of BSA surface modification of biotinylated *E. coli*

The successful conjugation of biotinylated *E. coli* to streptavidin-labeled BSA was detected by immunofluorescence experiments using appropriate anti-streptavidin primary antibodies. 20 µl of biotinylated *E. coli*-conjugated BSA mixture (*E. coli*-BSA) was dropped onto 0.1 mg/ml L-polylysine solution-coated culture plate. *E. coli* bacteria were treated with 0.02% Triton x-100 (solarbio, Beijing) and 2.5% Glutaraldehyde (EM Grade) for 20 min, washed three times with PBS and dehydrated with methanol by natural evaporation to complete the attachment of *E. coli*. 10% goat serum was added for 1 h (without wash). The control group and the experimental group were incubated with primary antibody and PBS at 4℃ overnight. Sterile PBS was added thrice for 5 min each time and incubated at room temperature with the secondary antibody for 1 h. The was with sterile PBS was repeated thrice for 5 min each time, and the sealing solution containing DAPI was added, followed by microscopic observation of the sample after 30 min.

#### Adhesion and targeting ability of BSA modified *E. coli*

In order to observe the adhesion and targeting ability of tumor cells by *E. coli*-BSA, *E. coli*-BSA was co-cultured with different bladder cancer cell lines and the relative position and morphology of tumor cells and bacteria were observed.

##### EGFP plasmid transduction in E. coli

100 µl of competent cells was thawed on ice. 1 µl of 100 ng/µl pET-28a-EGFP-C was added to the competent cell suspension, and the centrifuge tube was gently swirled to mix the contents. After standing in the ice bath for 30 min, the centrifuge tube was placed in a 42 °C water bath for 60–90 s, then it was quickly transferred to the ice bath for 2–3 min. 500 µl sterile anti-antibiotic LB medium was added to the centrifuge tube, shaken at 180 rpm at 37 °C for 1 h. An appropriate amount of the transformed competent cells was taken and spread on LB plates containing kanamycin and the plates were cultured in an inverted fashion at 37 °C for 12–16 h. Following this, a single colony was picked and cultured to the logarithmic phase for subsequent use.

##### Detection of SPARC protein expression level

The *DepMap* [40] database was used to detect the expression of SPARC in different bladder cancer cell lines. 1 mL of different bladder cancer cells at 8 × 10^5^ cell/mL density was spread into a 6-well plate. After the plates were confluent, 600 µL of serum-free 1640 medium was added to the plates for 24 h, and then the supernatant was taken. According to the instructions of Boster, the supernatant was diluted two times with the sample diluent, and the amount of secreted SPARC protein in the supernatant was detected. High and low SPARC expressing bladder cancer cell lines were selected for DAPI staining and for verifying the adhesion and targeting ability of *E. coli*-BSA.

##### Detection of the adhesion ability of *E. coli* -BSA to tumor Cells

After co-culturing bladder cancer cells with *E. coli*-BSA or unmodified *E. coli* for 3 h, the free *E. coli* was washed away with PBS. The adhesion of unmodified *E. coli* and *E. coli*-BSA to bladder cancer cells was observed under fluorescence microscope (Olympus).

##### Detection of tumor cell targeting by *E. coli*-BSA

To detect the targeting ability of unmodified *E. coli* and *E. coli*-BSA to bladder cells, bladder cancer cells Tccsup and Scaber were co-cultured with *E. coli*-BSA or unmodified *E. coli* for 12 h. Then medium containing gentamicin was added to kill non-internalized *E. coli* and *E. coli*-BSA and incubated for 24 h at 37 degrees Celsius, 5% CO2. After washing away free *E. coli* with PBS, the adhesion of unmodified *E. coli* and *E. coli*-BSA to bladder cancer cells was observed under fluorescence microscope (Olympus).

## Results

### Detection of biotinylated *E. coli*

**Fig. 2:**
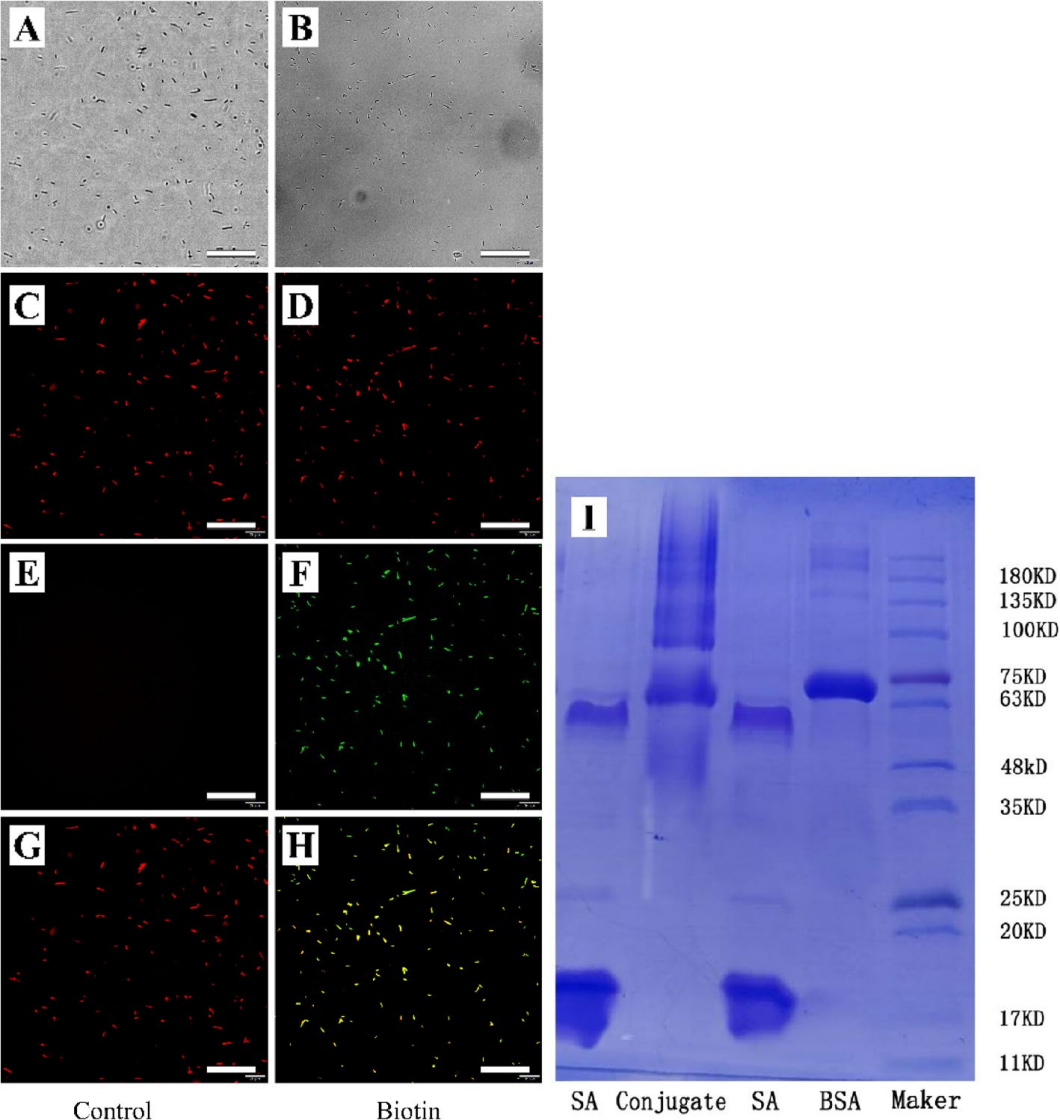
Images showing biotinylated *E. coli* under inverted fluorescence microscope** A-B**: bright field; **C**-**D**: PI co-localized red fluorescence;** E–F**: green fluorescence combined with streptavidin** G**-**H**: B and C merged (Scale bar = 50 µm A-H) **I**: BSA conjugated streptavidin monomer SA: streptavidin; Conjugate: BSA- streptavidin

Biotinylated and non-biotinylated *E. coli* reacted with streptavidin could be detected by green fluorescence at 488 nm after 30 min incubation on ice. The co-localization was confirmed by staining with PI. *E. coli* could be seen in both the groups in the bright field and PI co-localization staining could be seen in the visual field (Fig. 2A-D). Obvious green fluorescence was observed when biotinylated *E. coli* conjugated with streptavidin in the experimental group (Fig. 2F), while no green fluorescence was seen in the control group without biotinylated *E. coli* (Fig. 2E). Figure 2G is the merge of Fig. 2C and E, Fig. 2H is the merge of Fig. 2D and F.

### Detection of streptavidin-conjugated BSA

Streptavidin-conjugated BSA was detected by non-reducing protein gel electrophoresis and Coomassie brilliant blue staining. The molecular weight of BSA is 66 KD, and the molecular weight of streptavidin tetramer is 58 KD, which is coupled with one or more streptavidin monomers to form a drag band on the conjugate lane (Fig. 2I).

### Detection of biotinylated *E. coli* and streptavidin-labeled BSA

**Fig. 3:**
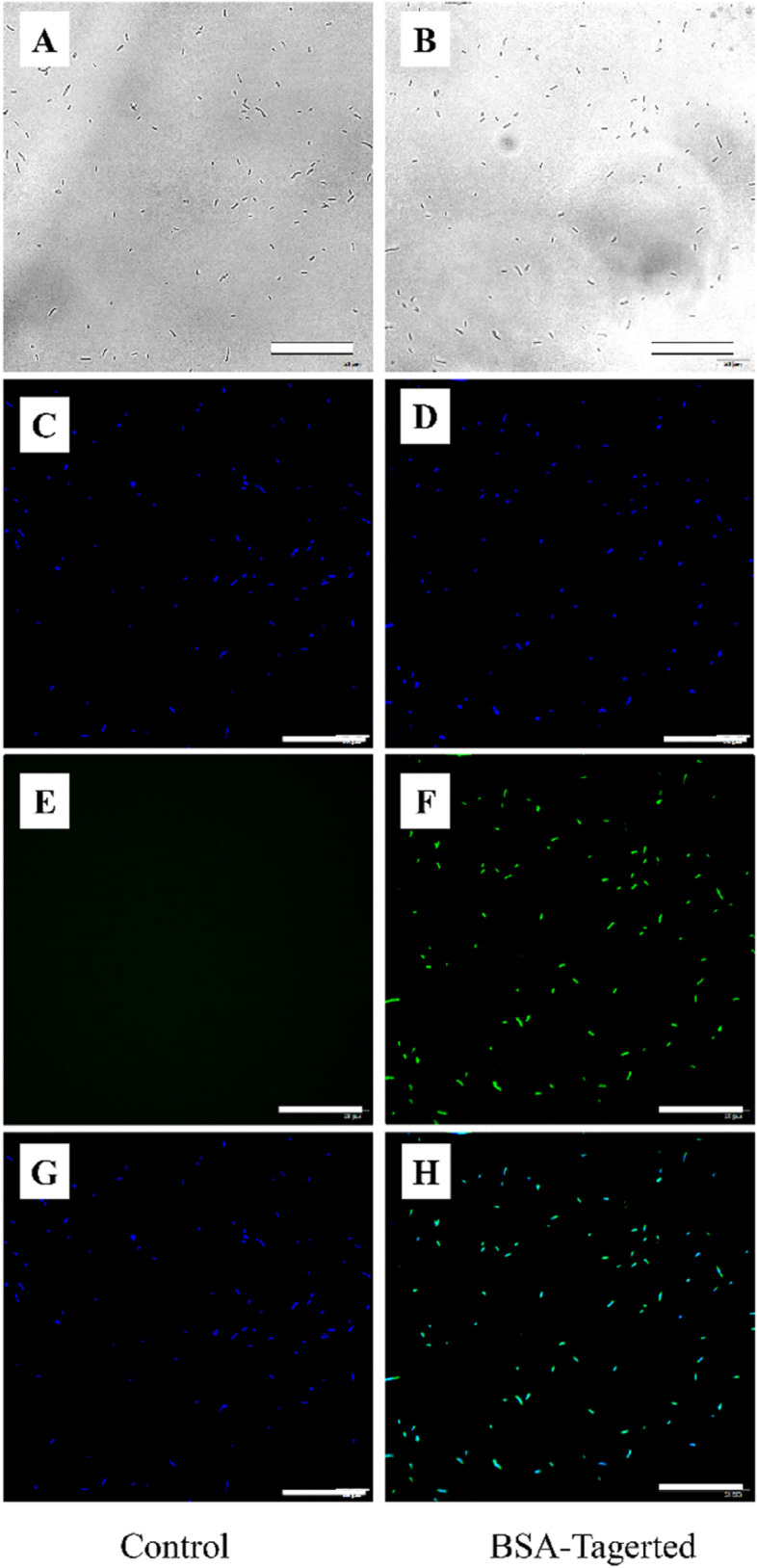
Images of *E. coli*-BSA under inverted fluorescence microscope: Experimental group: anti-streptavidin primary antibody immunofluorescence; Control group: PBS instead of anti-streptavidin primary antibody; **A-B**: bright field; **C-D**: DAPI co-localization fluorescence image; **E–F**: FITC-labeled streptavidin fluorescence; **G-H**: B and C merged (Scale bar = 50 µm A-H)

After the coupling of biotinylated *E. coli* with streptavidin labeled BSA was completed, normal streptavidin primary antibody was used in the experimental group and PBS was used in the control group. Immunofluorescence staining was performed under the same experimental conditions. *E. coli* could be seen in the bright field (Fig. 3A, B) and DAPI co-localization staining was observed in both the groups (Fig. 3C, D). In the experimental group, the biotinylated *E. coli* was successfully conjugated to streptavidin-coupled BSA. Streptavidin could be detected by the obvious green fluorescence (Fig. 3F), while no green fluorescence was found in the control group without biotin conjugation (Fig. 3E). Figure 3G shows the merge of Fig. 3C and E, Fig. 3H shows the merge of Fig. 3D and F.

### Detection of targeting and adhesion of *E. coli*-BSA to bladder cancer cells

#### Detection of SPARC expression

**Fig. 4:**
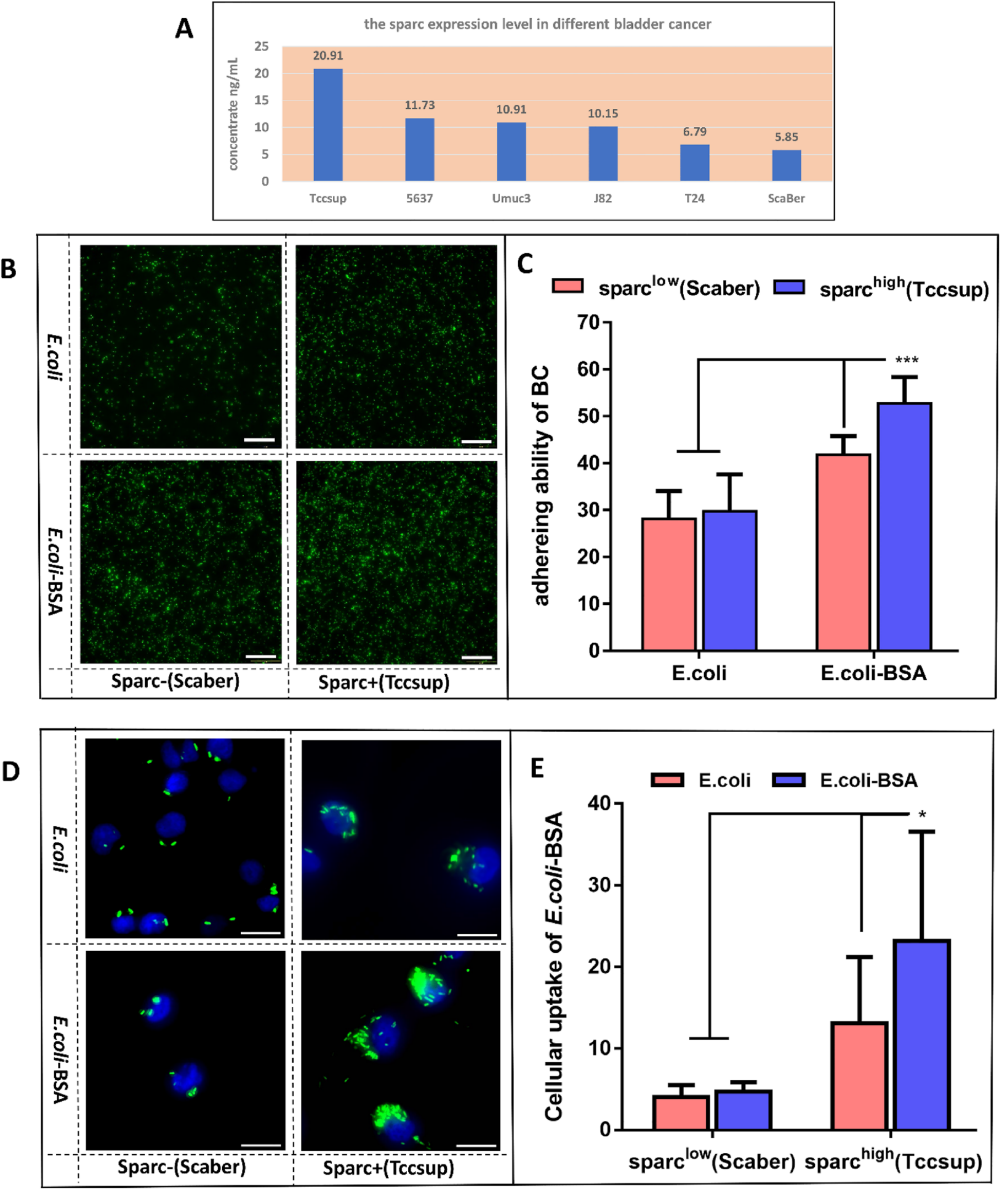
Detection of SPARC expression levels in different bladder cancer cell lines. **A** The amount of secretion SPARC in the supernatant of different bladder cancer cell lines. **B** and **C** Comparison of the adhesion ability of unmodified *E. coli* and *E. coli*-BSA to Tccsup and Scaber cells. **B** Fluorescence microscope images (Scale bar = 100 µm), **C** Image analysis of cellular uptake in panel B was quantified using Image J. *** indicates *p* < 0.005. **D** and **E** Comparison of the targeting ability of unmodified *E. coli* and *E. coli*-BSA to Tccsup and Scaber cells. **D** Fluorescence microscope images (Scale bar = 20 µm). **E** Image analysis of cell uptake in panel D was quantified using Image J. * indicates *p* < 0.05

In the DepMap database, we searched bladder cancer cell lines with different SPARC expression levels. After culturing the respective bladder cancer cells for 36 h, the culture supernatant was taken, and the expression levels of SPARC was measured in the supernatant according to the instructions of the Boster SPARC ELISA kit. Based on the results (Fig. 4A), the cell lines Tccsup and Scaber were selected for further experiments.

#### Detection of the adhesion of *E. coli*-BSA to bladder cancer cells

After co-culturing unmodified *E. coli* or *E. coli*-BSA with bladder cancer cells for 2 h, free *E. coli* was washed away with PBS. Compared with unmodified *E. coli*, *E. coli*-BSA showed stronger adhesion to Tccsup cells, but no statistically significant difference was observed in Scaber cells (Fig. 4B, C). Thus, compared with Scaber cells, *E. coli*-BSA showed stronger ability to adhere to Tccsup cells (Fig. 4B, C).

#### Detection of the tumor cells targeting ability of *E. coli*-BSA in vitro

Bladder cancer cells Tccsup, Scaber were co-cultured with unmodified *E. coli* or *E. coli*-BSA for 12 h. Fluorescent microscope observation showed that *E. coli*-BSA had stronger ability to target Tccsup cells than Scaber cells (Fig. 4D, E). Compared with unmodified *E. coli*, *E. coli*-BSA had a stronger ability to target Tccup cells, but there was no statistically significant difference between the two in Scaber cells. (Fig. 4D,E).

## Discussion

Several new anti-cancer therapeutics have been developed to overcome the limitation of conventional agents Among them, live bacteria therapy and medical BSA materials have attracted much attention due to their respective advantages for treating cancer. Our study used the high affinity between biotin and streptavidin to modify the surface of *E. coli* with BSA, which enhanced the adhesion and targeting of *E. coli* to bladder cancer cell lines. Moreover, we found that this adhesion and targeting were associated with the abundance of SPARC expression in bladder cancer cells.

In biotinylated *E. coli*, due to the difference in primary amine density, surface charge and total surface area between *E. coli*, there may be an impact on the coupling efficiency [41, 42]. This may lead to only a small amount of *E. coli* to be coupled to biotin, resulting in a partial difference in the fluorescence signal produced by *E. coli* when biotinylated *E. coli* biotinylation was verified by streptavidin with green fluorescence. When the denaturing and non-reducing reaction of streptavidin coupled BSA was carried out, the purity of BSA was insufficient, which led to the residual shadow above the main band. Due to pH, protein isoelectric point and other potential factors, the streptavidin tetramer (M = 58KD) could not be completely dissociated, so the separation phenomenon of streptavidin tetramer and polymer appeared in the figure (Fig. 2I). Nevertheless, the molecular weight of the mixture of streptavidin conjugated BSA showed that BSA with a molecular weight of 66 KD was successfully coupled with the dissociated streptavidin monomer, and the number of BSA molecules bound to a streptavidin monomer was the highest.

After identifying that *E. coli*-BSA enhanced the targeting and adhesion of *E. coli* to bladder cancer cells, we further explored the underlying mechanism. Compared with unmodified *E. coli* and cell lines with low expression of SPARC protein, *E. coli*-BSA had stronger adhesion and targeting ability to bladder cancer cells with high expression of SPARC, but there is no significant difference in whether *E. coli* is modified in bladder cancer cell lines with low SPARC expression. The above differences indicated that the effect of BSA-modified *E. coli* on the adhesion and targeting of bladder cancer cells may be related to the expression of SPARC protein in bladder cancer cells. We found a significant correlation of the increased adhesion of *E. coli* to bladder cancer cells and the BSA modification, however, the correlation of adhesion ability of *E. coli* was stronger to the level of SPARC expression in the bladder cancer cells. Thus, both the surface modification of *E. coli* and expression of SPARC in the cancer cells, could alter the targeting and adhesion abilities of *E. coli*. However, there may be other factors influencing the adhesion ability of *E. coli*. Thus, for bladder cancer patients with high SPARC expression, on the basis of BSA modification of live bacteria, combination with other therapeutics may provide an effective therapeutic strategy.

A positive immunofluorescence signal from streptavidin indicated the successful completion of surface BSA modification of *E. coli*. Consistent with this, Mostaghaci et al. reported in 2017 that the synthetic particles modified by biotin-streptavidin binding on the surface of bacteria were able to attach more effectively to urinary tract epithelial cells and gastrointestinal epithelial cells because of their affinity to mannose molecules expressed on the cell surface [43]. Vargason and Anselmo et al. achieved the same results in 2020 when adhesin antibody molecules were modified on the surfaces of *Lactobacillus casei* (LC), *E. coli* (EC) and *Bacillus coagulans* (BC). It was also shown that before and after modification, the growth capacity and activity of bacteria remained unchanged, but their adhesion to gastrointestinal tract and resistance to the colonization of pathogenic bacteria were enhanced [41, 42]. What is more gratifying is that when we co-incubated *E. coli*-BSA with Tccsup and 5637 bladder cancer cells expressing higher levels of SPARC, the Tccsup cells were infected by a high number of *E. coli-BSA* after 12 h (Additional file). These data not only provide feasibility support for the bacterial surface BSA modification strategy, but also reveal the anti-cancer potential of such a strategy. In particular, certain solid tumors with special anatomical location can be administered by intratumoral, subcutaneous injection, perfusion, oral administration. Furthermore, our novel strategy could prevent bacteria-protein conjugates from being recognized and cleared by human liver, kidney, lysosome and complex immune system when transported to the tumor site [25, 44, 45].

The complexity of bacteria as organisms determines the difficulty and risk of turning them into anti-cancer weapons. However, complexity is precisely the most fascinating part of bacteria. Different species of bacteria have different biological, physical and chemical characteristics. Bacteria in tumors can have complex effects on tumor progression or inhibition by affecting different immune pathways, resulting in diverse anti-cancer effects after their surface modification [46–48]. This allows scientists to fine-tune the functions of different strains to achieve anti-cancer activity that other treatments cannot achieve [49]. Fortunately, most of the easily modifiable engineered strains contain primary amine groups, which provides essential conditions for surface BSA modification and anti-cancer application of engineered bacteria. At present, a variety of bacterial therapies have been successfully implemented in humans and have entered phase I/II clinical trials [50, 51]. In these clinical trials, bacteria were mostly used as anti-cancer agents carrying cytotoxic proteins, cytokines, angiogenesis inhibitors, antigens and antibody. Despite the complex pathophysiological characteristics of tumor and the development of bacterial therapy, traditional anti-cancer methods such as surgery, radiotherapy and chemotherapy are still the current first-line anti-cancer choice. The bacterial vector has achieved gratifying results in the experimental model, there are still some challenges in assessing the safety, effectiveness and accuracy of these agents, and further research is needed to evaluate its efficacy and toxicity in the treatment of cancer in the clinic. Although BSA is popular as a new type of biological research material, compared with human serum albumin (HAS), its immune anti-host response will also become a huge challenge in its research and development [21, 23]. The combination of bacterial therapy and conventional therapy has shown better therapeutic efficacy and disease prognosis and provides a better treatment for patients with cancer pedigree. In the future, many traditional single treatments will be replaced by multidisciplinary and multidirectional combination therapy. BSA modification on the surface of *E. coli* helps to resolve the instability of free BSA in liquid media. It also improves the targeting capabilities of the combination of bacteria and BSA while maintaining many desired physical and chemical properties such as biocompatibility and biodegradability. Utilizing the respective therapeutic advantages of existing bacteria and BSA may be beneficial and effective to break through the complex human immunity, vasculature, and various tumor immunity and evasion mechanisms mediated by the tumor microenvironment and its matrix. The proposed strategy would help the bacteria to reach and colonize in the hypoxic and necrotic core of the tumor, further triggering an immune response and this may provide the basis for subsequent drug delivery and tumor targeted therapy.

## Conclusion

Taking advantage of the respective anti-cancer properties of medical BSA materials and attenuated live bacteria therapy, we modified the surface of *E. coli* with BSA. In addition, to maintaining the biocompatibility, biodegradability and many other desirable physical and chemical properties, it also solved the problem of low aqueous solubility of free BSA. Moreover, we found that BSA-modified *E. coli* had enhanced adhesion and tumor targeting ability, which may be related to the expression of the BSA-binding protein SPARC in bladder cancer cells. Our findings may pave the way for future studies exploring the combination of BSA with live bacteria and expand the application of live bacteria in the treatment of different types of cancer.

## Supplementary Information

**Additional file 1.**

## Data Availability

The datasets generated and/or analyzed during the current study are available from the corresponding author upon reasonable request.
