# Supplementary material for: BSA modification of bacterial surface: a promising anti-cancer therapeutic strategy
Source: BMC Microbiol. 2023 Apr 17;23:105. doi: 10.1186/s12866-023-02830-z (PMC10108468; doi:10.1186/s12866-023-02830-z)
Supplement: Supplementary file 1 — Additional file 1. [file 12866_2023_2830_MOESM1_ESM.zip › Supplementary Information.docx]

**Supplementary Information**

**Additional file 1 and 2: 5637(Turn Around X and Y):** Tccsup was infected by *E. coli-BSA*.

**Additional file 3 and 4: Tccsup-z-position (1) and (2):** Tccsup was infected by *E. coli-BSA*.

**Additional file 5: Supplementary file1:** The full-length gels and blots and notes……..….1

**Additional file 6: Supplementary file2:** Notes to Additional file 1 to 4……………..……2-3

**Additional file 5: Supplementary file1:** The full-length gels and blots and notes


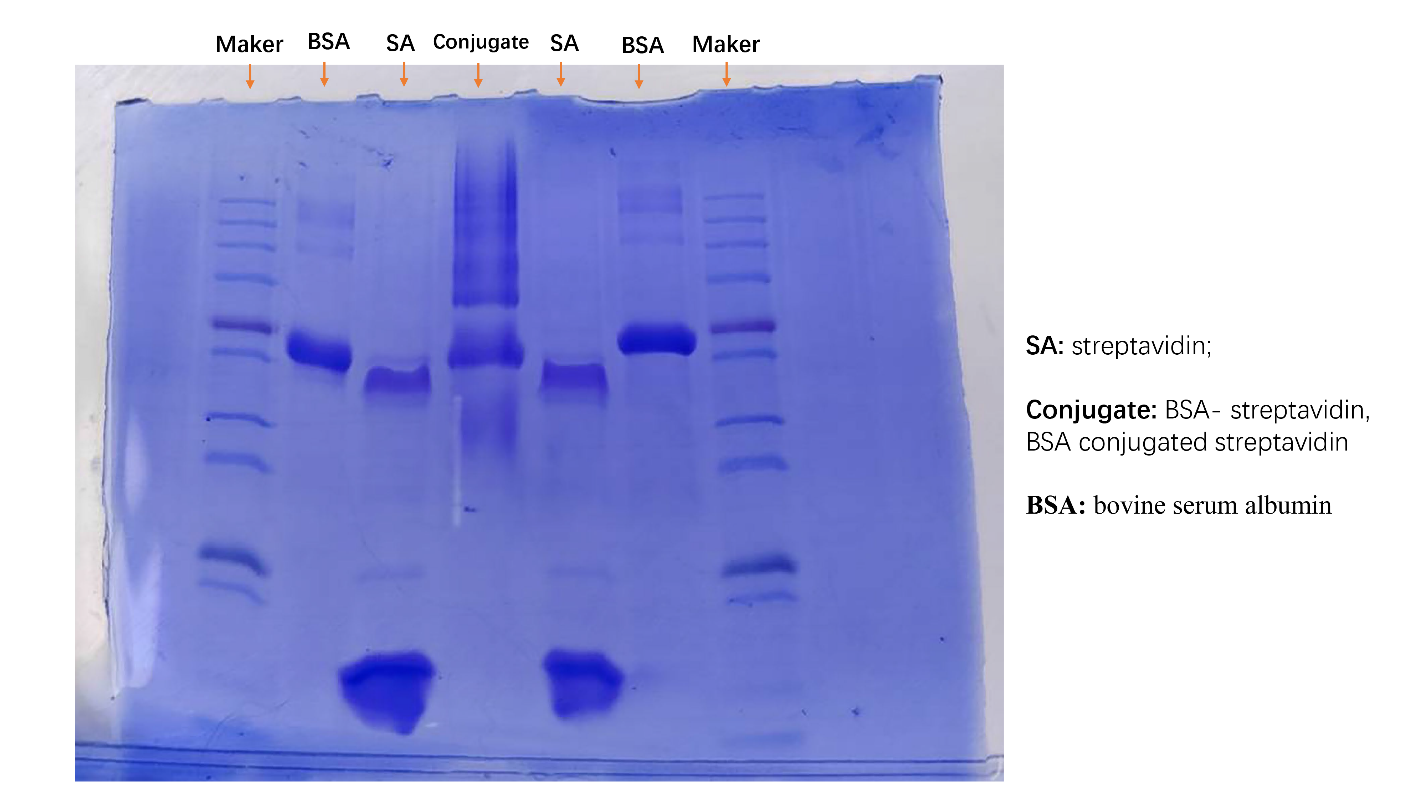


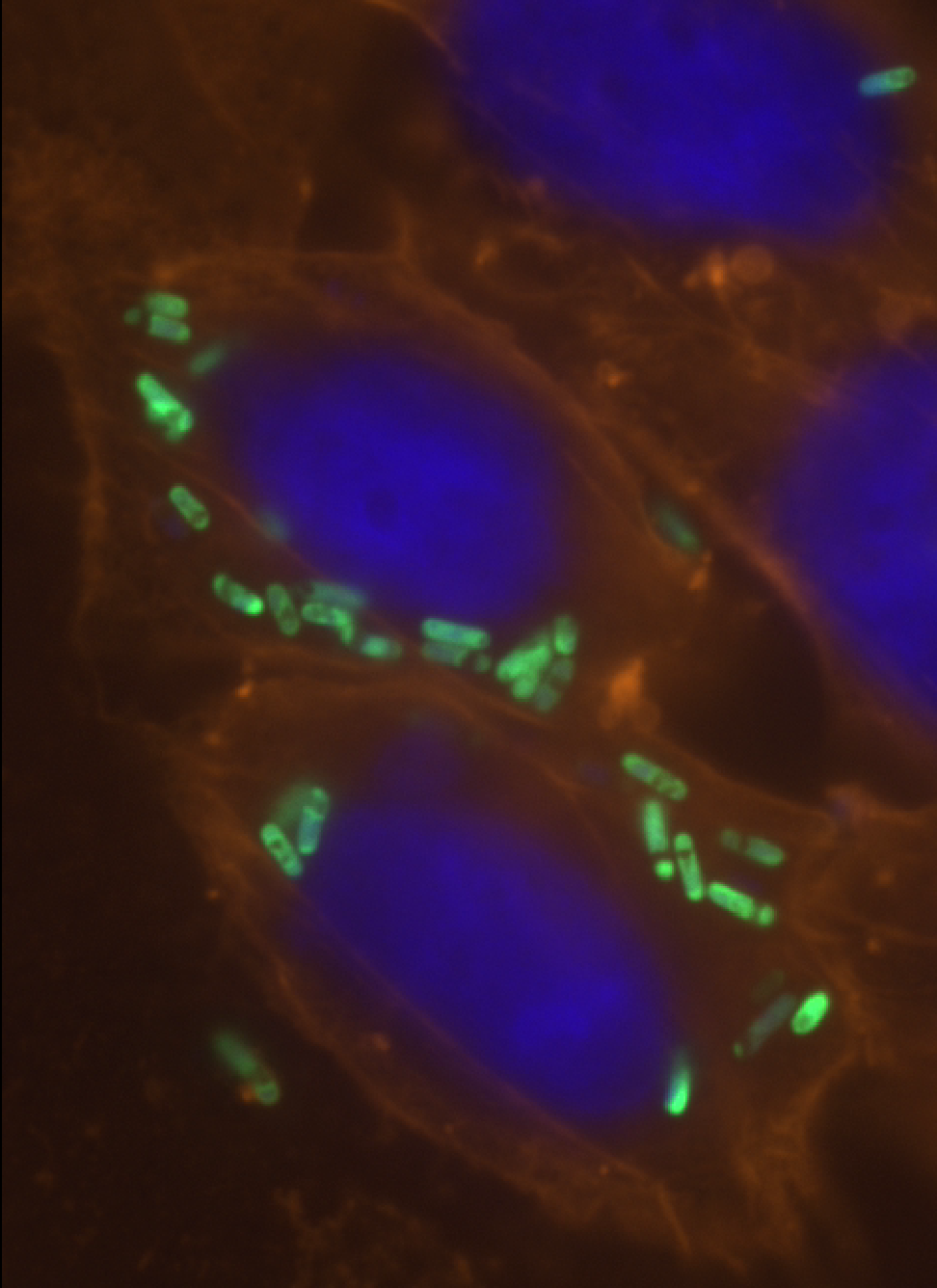


**3**

**2**

**1**


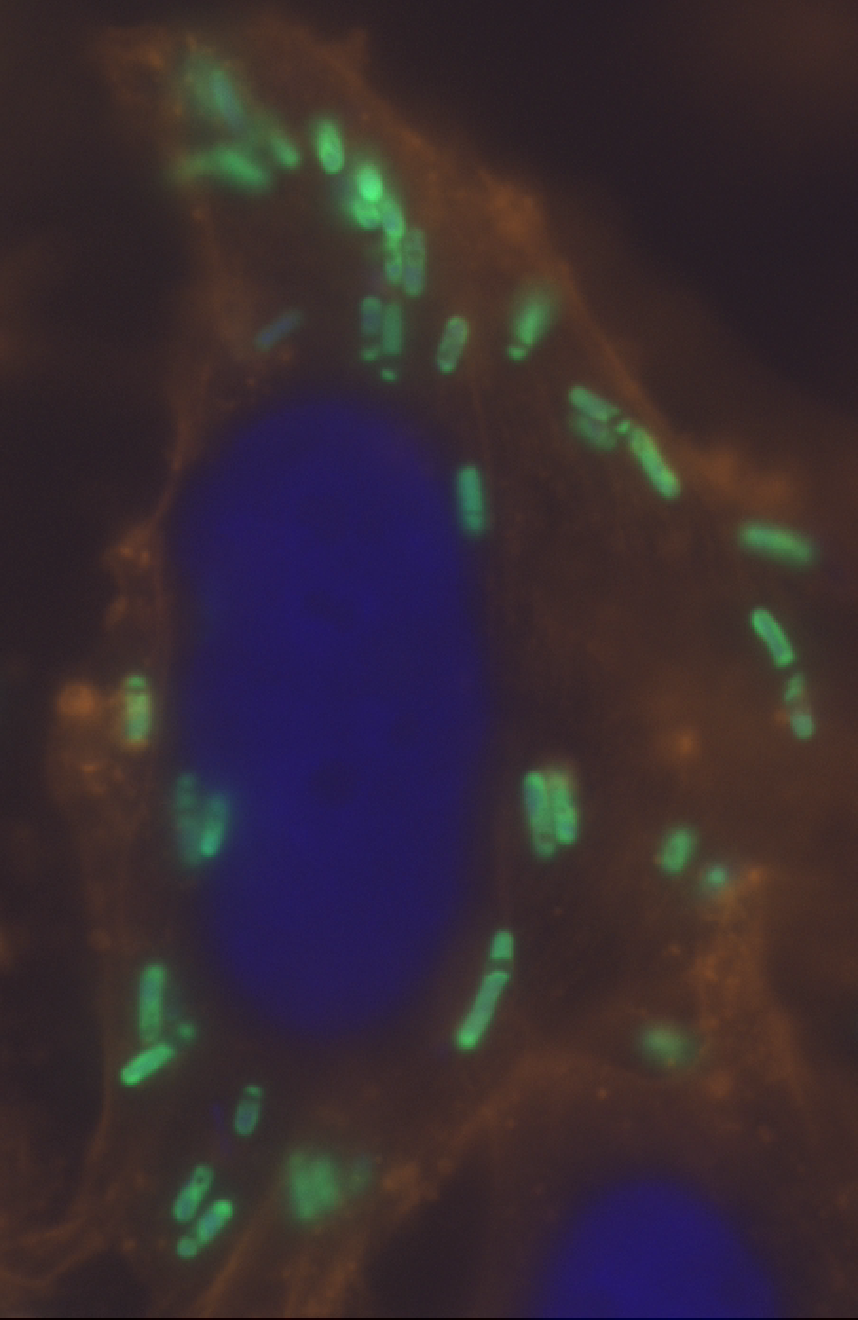


**3**

**2**

**1**

**Notes to Supplementary Tccsup-z-position (1) and (2)**

**1.** nucleus

**2.** *E.coli*-BSA

**3.** Cytoplasm


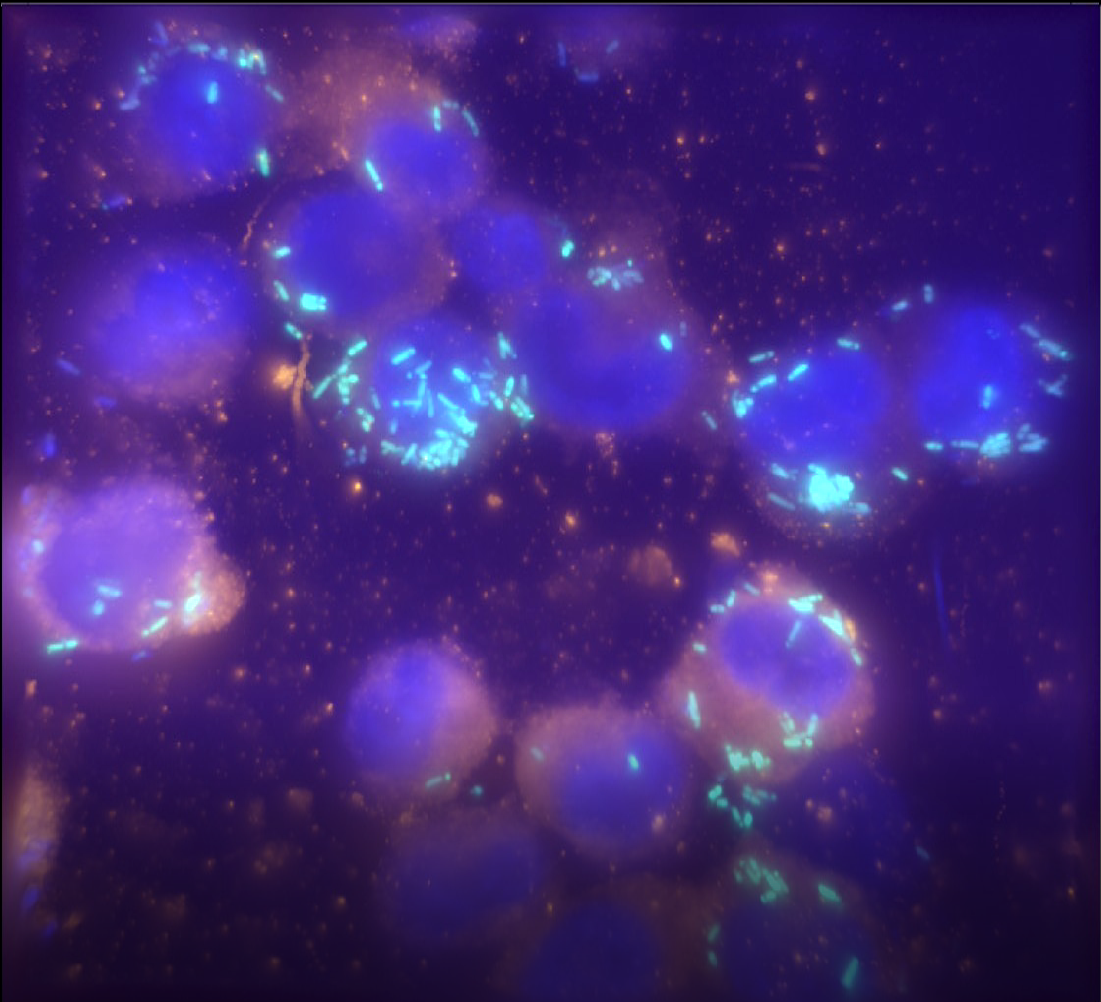


**3**

**2**

**1**

**Notes to Supplementary 5637 (Turn Around X and Y)**

**1.** nucleus

**2.** *E.coli*-BSA

**3.** Cytoplasm
